# Cartas al editor

**DOI:** 10.7705/biomedica.7747

**Published:** 2024-11-06

Bogotá, D. C., 22 de septiembre de 2024

Señores

Comité Editorial de *Biomédica*

La ciudad

Estimados señores:

Reciban un cordial saludo.

Les escribo porque para mi tesis de maestría estoy haciendo una revisión de la literatura y al detallar el artículo “Factores asociados al tratamiento no exitoso para tuberculosis en pacientes previamente tratados en Cali, Colombia, en el periodo 2015 -2019” (Biomédica, 2023;43360-73. https://doi.org/10.7705/biomedica.6961), encontré una inconsistencia que merece ser revisada por parte de la revista *Biomédica*, donde se encuentra publicado.

Específicamente, en el apartado de la discusión, los autores citan la referencia 37 en las siguientes líneas: “Un estudio en Armenia (Colombia) de pacientes con tuberculosis, independientemente de su estado de ingreso, reportó que la probabilidad de fracasar con el tratamiento fue 8,99 veces mayor en aquellos con diabetes que en aquellos sin diabetes (37)”.

*37. Sahakyan S, Petrosyan V, Abrahamyan L. Diabetes mellitus and treatment outcomes of pulmonary tuberculosis: A cohort study. Int J Public Health. 2020;65:37-43.**https://doi.org/10.1007/s00038-019-01277-2*

Los autores mencionan que dicha investigación fue hecha en Armenia (Colombia), lo cual es incorrecto, ya que el estudio citado corresponde a Armenia, el país de Asia, y, más específicamente, a Yerevan, capital de Armenia.

Considero importante que se realice el ajuste en el artículo y se aclare que el dato no corresponde a una investigación en Colombia, ya que podría generar interpretaciones erróneas de los resultados.

Heidy Natalia Urrego Parra

Maestría en Salud Pública, Facultad de Medicina, Universidad Nacional de Colombia
