# Supplementary material for: Cartas al editor
Source: Biomedica. 2024 Nov 6;44(4):575–8. [Article in Spanish] doi: 10.7705/biomedica.7747 (PMC11670869; doi:10.7705/biomedica.7747)
Supplement: Related texts

# Reply: Cartas al Editor

Señores

Comité Editorial de *Biomédica* Bogotá, D. C.

Estimados señores:

Reciban un cordial saludo.

Agradecemos la lectura crítica de nuestra investigación, así como la oportunidad de profundizar en nuestros hallazgos, en especial en aquellos relacionados con personas que luchan contra la diabetes y la tuberculosis de manera concomitante. A nivel mundial, la prevalencia de diabetes en pacientes con tuberculosis alcanza el 15,3 % ^1^ e incrementa el riesgo de desenlaces adversos como fracaso terapéutico, muerte y recaída por tuberculosis ^2^.

En nuestro estudio, realizado en una cohorte retrospectiva de pacientes que reingresaron al programa de micobacterias en Cali (Colombia), informamos que el tener diabetes se asoció de manera independiente con el fracaso del tratamiento para tuberculosis en personas con antecedentes de tratamiento para esta última (ORa = 1,89; IC_95%_: 1,29 - 2,77) ^3^. En la sección de discusión, comparamos este hallazgo con lo informado en el estudio de Sahakyan et al. ^4^, máxime al ser realizados en localizaciones geográficas diferentes. Estudios en otras regiones presentaron resultados similares en cuanto a la diabetes y el tratamiento para la tuberculosis ^2^^-^^5^. En vista de la carga elevada de ambas enfermedades, varios autores recomiendan que los programas de control de tuberculosis fortalezcan su capacidad para el manejo integral coordinado y el seguimiento estrecho de pacientes con comorbilidades, particularmente aquellos con diabetes ^2^^,^^6^^-^^8^.

Las personas con diabetes se benefician del tratamiento y seguimiento médico regular para mantener el control glucémico y gestionar los riesgos cardiovasculares ^9^. La tuberculosis, que afecta usualmente a personas con vulnerabilidad socioeconómica, empeora el control glucémico y también exige un esquema farmacológico continuo por un periodo relativamente prolongado y que requiere de una observancia apropiada. En Colombia, para la tuberculosis pulmonar sensible se emplea un esquema de cuatro medicamentos (isoniacida, rifampicina, pirazinamida y etambutol) administrados casi todos los días durante dos meses, aproximadamente, seguido de un esquema de isoniacida y rifampicina, aproximadamente, por cuatro meses más ^10^. En ese sentido, la adhesión al tratamiento para la tuberculosis ya es de por sí desafiante, pero lo es aún más cuando se requieren controles y tratamientos médicos regulares para otras afecciones concomitantes como la diabetes, especialmente para personas que usualmente enfrentan vulnerabilidades socioeconómicas y están en un entorno de atención en salud fragmentado ^7^^,^^11^.

En Colombia, bajo el Sistema General de Seguridad Social en Salud, las atenciones son segmentadas por tipo de régimen de salud y condiciones económicas que permiten el coste de planes complementarios y medicina prepagada. Aunque más del 50 % de la población está subsidiada por el Estado (lo que permite que el nivel de aseguramiento sea elevado), el acceso efectivo a los servicios de salud es limitado ^12^. Cada entidad administradora de planes de beneficios de salud organiza su red de prestadores según las condiciones del mercado, lo que puede obligar a los usuarios a desplazarse largas distancias o de un lado a otro para conseguir que le autoricen procedimientos, recibir atención médica o someterse a exámenes diagnósticos, lo que afecta la continuidad de la atención y la adhesión al manejo médico ^12^.

Además, la fragmentación de la atención de los pacientes con diabetes y tuberculosis dificulta la coordinación entre especialidades y el manejo de comorbilidades y puede llevar a peores resultados de salud ^6^. En lugares de ingresos bajos y medios, donde la carga de ambas enfermedades es considerable, esta fragmentación puede resultar en diagnósticos tardíos y tratamientos inoportunos o a la falta de ellos ^11^. Por otra parte, el trabajo coordinado de servicios de atención para la diabetes y la tuberculosis puede mejorar el cumplimiento del tratamiento, facilitar el cribado bidireccional y reducir la pérdida de seguimiento ^9^^,^^11^. Sin embargo, para implementar efectivamente estrategias de manejo integradas para diabetes u otras enfermedades crónicas no transmisibles y tuberculosis es necesario abordar barreras, como la falta de recursos, la capacitación insuficiente del personal y la falta de conciencia sobre la coexistencia de estas enfermedades ^6^^-^^8^.

En este sentido, la colaboración entre los programas de diabetes y tuberculosis sería clave para superar estos desafíos y optimizar los recursos disponibles ^6^^,^^9^^,^^11^. Aunque se han promovido recomendaciones para la integración de la atención de la tuberculosis y las enfermedades no transmisibles, la implementación de esta estrategia es compleja y aún se necesita más investigación ^6^^,^^7^^,^^13^. Mientras se avanza hacia una coordinación efectiva entre los programas de enfermedades infecciosas y crónicas no transmisibles, resulta fundamental identificar, de manera temprana, las poblaciones que podrían beneficiarse de un seguimiento más estrecho y coordinado con el objetivo de reducir las barreras de acceso y mejorar la adhesión al tratamiento ^6^.

Ahora bien, respecto a la observación realizada sobre la ubicación geográfica de un estudio referenciado, aclaramos que el error se limita a la precisión de la localización (Armenia, país). Esto no afecta los resultados ni las conclusiones de nuestro estudio. En concreto, Sahakyan et al. ^4^, informaron que, independientemente del estado de ingreso de los pacientes, aquellos con diabetes tuvieron un riesgo 8,99 veces mayor de fracaso en el tratamiento de la tuberculosis. Este hallazgo fue citado para ilustrar la consistencia de los resultados documentada en otros contextos geográficos, lo que refuerza la validez de nuestra observación sobre la asociación entre diabetes y el fracaso del tratamiento de tuberculosis ^3^.

Si bien el señalar que Armenia se refería a la ciudad de Colombia en lugar del país pudo generar confusión en el lector, el contenido científico y el contexto sigue siendo válido y pertinente para el argumento presentado -centrado en la asociación de la diabetes y los malos resultados con el tratamiento para tuberculosis-. Hemos tomado medidas para corregir este tipo de errores en futuras publicaciones, agradeciendo la atención a este detalle por parte del lector. Recalcamos que dicha imprecisión no afecta la validez interna o externa de nuestros resultados, especialmente porque la coherencia de los hallazgos de nuestro estudio con otros de diferentes regiones -especialmente internacionales- refuerza la validez externa de la asociación independiente entre la diabetes con el fracaso del tratamiento para tuberculosis, expuesta en nuestro estudio. Además, señalamos que los lectores pueden ver y consultar la referencia citada con el enlace directo de consulta en el mismo párrafo y, por lo tanto, realizar la debida verificación de los datos.

Con lo anterior, esperamos haber dado una respuesta de fondo a la observación. Agradecemos la oportunidad de responder al lector y discutir con mayor amplitud nuestros hallazgos.

Robinson Pacheco López, José F. Fuertes-Bucheli, Liddy Varela y Jimena Ortiz

## References

[1] Noubiap JJ, Nansseu JR, Nyaga UF, Nkeck JR, Endomba FT, Kaze AD (2019). Global prevalence of diabetes in active tuberculosis: A systematic review and meta-analysis of data from 2.3 million patients with tuberculosis. Lancet Glob Health.

[2] BakerMA Harries AD, Jeon CY HartJE, Kapur A Lõnnroth K (2011). The impactofdiabetes on tuberculosis treatment outcomes: A systematic review. BMC Med.

[3] Varela L, Ortiz J, García PK, Luna L, Fuertes-Bucheli JF, Pacheco R. (2023). Factores asociados al tratamiento no exitoso para tuberculosis en pacientes previamente tratados en Cali, Colombia, en el periodo 2015-2019. Biomédica.

[4] Sahakyan S, Petrosyan V, Abrahamyan L. (2020). Diabetes mellitus and treatment outcomes of pulmonary tuberculosis: A cohort study. Int J Public Health.

[5] Gautam S, Shrestha N, Mahato S, Nguyen TPA, Mishra SR, Berg-Beckhoff G. (2021). Diabetes among tuberculosis patients and its impact on tuberculosis treatment in South Asia: A systematic review and meta-analysis. Scientific Reports.

[6] De Foo C, Shrestha P, Wang L, Du Q, García Basteiro AL, Abdullah AS (2022). Integrating tuberculosis and noncommunicable diseases care in low- and middle-income countries (LMICs): A systematic review. PLoS Med.

[7] Workneh MH, Bjune GA, Yimer SA. (2016). Assessment of health system challenges and opportunities for possible integration of diabetes mellitus and tuberculosis services in South- Eastern Amhara Region, Ethiopia: A qualitative study. BMC Health Serv Res.

[8] Shayo FK, Shayo SC. (2019). Availability and readiness of diabetes health facilities to manage tuberculosis in Tanzania: A path towards integrating tuberculosis-diabetes services in a high burden setting?. BMC Public Health.

[9] American Diabetes Association Professional Practice Committee (2024). 4. Comprehensive Medical Evaluation and Assessment of Comorbidities: Standards of Care in Diabetes, 2024. Diabetes Care.

[10] Colombia, Ministerio de Salud y Protección Social (2020). Lineamiento técnico operativo del Programa Nacional de Tuberculosis, Resolución 227 del 2020.

[11] Goldhaber-Fiebert JD, Jeon CY, Cohen T, Murray MB. (2011). Diabetes mellitus and tuberculosis in countries with high tuberculosis burdens: Individual risks and social determinants. Int J Epidemiol.

[12] Organización Panamericana de la Salud (2012). Salud en las Américas edición de 2012. Panorama regional y perfiles de país.

[13] Pan American Health Organization (2017). Health in the Americas+, 2017 ed..
